# Predictors of resilience in older adults with lower limb osteoarthritis and persistent severe pain

**DOI:** 10.1186/s12877-022-02926-7

**Published:** 2022-03-24

**Authors:** Natasja M. van Schoor, Erik J. Timmermans, Martijn Huisman, Alicia Gutiérrez-Misis, Willem Lems, Elaine M. Dennison, Maria Victoria Castell, Michael D. Denkinger, Nancy L. Pedersen, Stefania Maggi, Dorly J. H. Deeg

**Affiliations:** 1 Department of Epidemiology and Data Science, UMC, Vrije Universiteit Amsterdam, Amsterdam Public Health Research Institute, De Boelelaan 1117, 1081 HV Amsterdam, Netherlands; 2 Department of Sociology, Faculty of Social Sciences, Vrije Universiteit Amsterdam, Amsterdam, the Netherlands; 3 Medicine Department, Family Medicine and Primary Care Division, School of Medicine, Autonomous University of Madrid, Madrid, Spain; 4 Hospital La Paz Institute for Health Research (IdiPAZ), Madrid, Spain; 5 Department of Rheumatology, Amsterdam UMC, Amsterdam, the Netherlands; 6 MRC Lifecourse Epidemiology Unit, University of Southampton, Southampton General Hospital, Southampton, UK; 7 CS Dr. Castroviejo. Primary Care (SERMAS). Medicine Department, Family Medicine and Primary Care Division, School of Medicine, Autonomous University of Madrid, Madrid, Spain; 8 Geriatric Research Unit and Geriatric Center, Agaplesion Bethesda Hospital, University of Ulm, Ulm, Germany; 9 Department of Medical Epidemiology and Biostatistics, Karolinska Institute, Stockholm, Sweden; 10 National Research Council, Neuroscience Institute, Padua, Italy

**Keywords:** Osteoarthritis, Pain, Resilience, Predictors, Older Adults

## Abstract

**Background:**

Resilience refers to the process in which people function well despite adversity. Persistent severe pain may be considered an adversity in people with lower limb osteoarthritis (LLOA). The objectives of this study are: (1) to identify what proportion of older adults with LLOA and persistent severe pain show good functioning; and (2) to explore predictors of resilience.

**Methods:**

Data from the European Project on OSteoArthritis (EPOSA) were used involving standardized data from six European population-based cohort studies. LLOA is defined as clinical knee and/or hip osteoarthritis. Persistent severe pain is defined as the highest tertile of the pain subscale of the Western Ontario and McMaster Universities Osteoarthritis Index both at baseline and follow-up. Resilience is defined as good physical, mental or social functioning at follow-up despite having LLOA with persistent severe pain.

**Results:**

In total, 95 (14.9%) out of 638 individuals with LLOA had persistent severe pain. Among these, 10 (11.0%), 54 (57.4%) and 49 (53.8%) had good physical, mental and social functioning, respectively. Only 4 individuals (4.5%) were resilient in all three domains of functioning. Younger age, male sex, higher education, higher mastery, smoking and alcohol use, higher physical activity levels, absence of chronic diseases, and more contacts with friends predicted resilience in one or more domains of functioning.

**Conclusions:**

Few people with LLOA and persistent severe pain showed good physical functioning and about half showed good mental or social functioning. Predictors of resilience differed between domains, and might provide new insights for treatment.

## Background

Osteoarthritis (OA) is the most common form of musculoskeletal disorders worldwide and an important contributor to global disability [1]. One of the key symptoms of clinical OA is pain [1]. In 2017, Bartley et al. indicated that most research up till then had been problem-focused in nature, emphasizing vulnerability and risk factors for pain [2]. They proposed that future research in OA should focus on factors that mollify the adverse effects of pain, which is also referred to as resilience research.

Resilience is a term used to refer to the process of effectively negotiating, adapting to, or managing significant sources of stress or trauma [3]. Research on resilience aims to explain why people thrive despite being exposed to specific risk experiences [4]. Resilience can be assessed by several different methods [5]. Two current methods are the use of a resilience scale and the use of a priori established criteria of resilience, i.e., by defining OA as the source of stress, and good quality of life as the resilience outcome [4, 5]. The former approach is based on a general conceptualisation of resilience as a psychological or psychosocial resource and has been studied as a determinant of OA-related outcomes such as knee pain, disability, and cellular aging [6–8]. An advantage of the latter approach, which will be used in the current study, is that the concept of resilience is customized to the specific situation of interest, and that it forces researchers to carefully select outcomes and protective factors that are relevant to the specific context of the stressor at hand [5]. As pain is one of the most disabling determinants in OA, we aim to study those people with clinical lower limb osteoarthritis (LLOA) who ‘thrive’, i.e. show good quality of life despite having persistent severe pain.

Our approach differs from previous literature, which mostly studied factors directly related to pain or disability, thereby focusing on one domain of quality of life only [9]. However, as older people are faced with declines in functioning in multiple domains during ageing, resilience research should not focus on one domain as it might be that people function well in one domain, but not in other domains of quality of life [4]. Conceptual models of quality of life and well-being indicate that generally two domains are important, i.e., physical and social functioning [10]. Pertinent domains may differ across individuals, as some people may attach greater value to a specific domain than others. Studies on lay views of quality of life underscore the importance of the physical and social domains, and add psychological well-being [11–13]. In recent literature, associations of LLOA with physical, mental and social functioning has been described [9, 14–16]. Therefore, in this study, resilience is defined as good physical, mental or social functioning at follow-up despite having LLOA with persistent severe pain.

The objectives of this study are: (1) to identify what proportion of older people with LLOA and persistent severe pain show good functioning in various domains of ageing; and (2) to explore predictors of resilience. For the second objective, a wide range of predictors is studied, including demographic (sex, age in years, educational level), personality (mastery), lifestyle (smoking, alcohol consumption, body mass index (BMI), physical activity, social conditions (partner status, social contacts) and health predictors (number of chronic diseases, grip strength).

## Materials and methods

### Design and study sample

Data from the European Project on OSteoArthritis (EPOSA) were used, which focuses on the personal and societal burden of OA and its determinants in community-dwelling older adults aged 65 to 85 years in six European countries. The study design and data collection of EPOSA are described elsewhere [17]. In short, random samples were taken from existing population-based cohorts in five European countries (Germany, the Netherlands, Spain, Sweden, and the United Kingdom). In Italy, a new sample was drawn. A total of 2,942 individuals was included at baseline. After 12–18 months, a follow-up measurement was performed in 2,455 individuals. For logistic reasons, follow-up duration differed slightly between individuals. In the current study, only people with LLOA at baseline were included (n = 638). Of these participants, 503 (78.8%) participated at follow-up. For all six countries, the study design and procedures were approved by the Ethical Review Boards of the respective institutions (GER: Ethikkommission Universität Ulm [22/11]; NL: Medisch Ethische Toetsingscommissie Vrije Universiteit Amsterdam [2002/141]; ES: Comité Ético de Investigación Clínica del Hospital Universitario La Paz Madrid [PI-1080]; SWE: Till forskningsetikkommittén vid Karolinska Instituted Stockholm [00–132]; UK: Hertfordshire Research Ethics Committee [10/H0311/59]; Italy: Comitato Etico Provinciale Treviso [XLIV-RSA/AULSS7]), and research was performed in compliance with the Helsinki Declaration. All participants provided written informed consent.

### Lower limb osteoarthritis

LLOA is defined as clinical hip and/or knee OA. Using self-report and physical examination data, algorithms based on the clinical classification criteria of the American College of Rheumatology were used to define clinical hip and knee OA [1, 18, 19]. Clinical hip OA was defined as: pain in the hip as evaluated by the Western Ontario and McMaster Universities Osteoarthritis Index (WOMAC) pain subscale score [20], plus all of: pain associated with hip internal rotation in at least one side; morning stiffness lasting < 60 min evaluated by the WOMAC stiffness subscale [20]; and over 50 years of age. Clinical knee OA was defined as: pain in the knee as evaluated by the WOMAC pain subscale score [20], plus any 3 of: morning stiffness lasting < 30 min evaluated by the WOMAC stiffness subscale [20]; crepitus on active motion in at least one side; bony tenderness in at least one side; bony enlargement in at least one side, no palpable warmth of synovium in both knees; and over 50 years of age.

### Pain severity

The WOMAC pain subscale contains five items with regard to pain in the knee and five items with regard to pain in the hip, respectively, experienced in the past 48 h: during walking on a flat surface, descending or ascending stairs, at night in bed, when sitting or lying, when standing. The responses to the five items on pain are scaled on a five point Likert scale ranging from none (score = 0) to extreme pain (score = 4). Missing values were imputed according to the user manual [20]. The scores were summed to get an overall pain score for knee and hip pain, respectively, and standardized to a score from 0 to 100. As knee and hip pain followed a slightly different distribution, knee and hip pain-specific thresholds were used. For hip OA, the highest tertile at baseline was a score of ≥ 40, and for knee OA, the highest tertile was a score of ≥ 35. At follow-up, the same cut-offs were used.

### Resilience

Resilience was defined as good physical, mental or social functioning at follow-up despite having LLOA with persistent severe pain, i.e., being in the highest tertile of the WOMAC pain subscale score both at baseline and follow-up. Physical functioning was assessed at follow-up using the WOMAC physical function subscale on difficulty doing 17 different daily activities. This concerned one set of questions asking about difficulties as a consequence of hip and/or knee problems. Missing values were imputed according to the user manual and subscale scores were standardized resulting in a subscale score ranging from 0 (no difficulties) to 100 (extreme difficulties) [20]. Good physical functioning was defined as present when an individual had a score ≤ 20.6, which was the median in our study.

Mental functioning was assessed at follow-up using the Hospital Anxiety Depression Scale (HADS). The HADS is a questionnaire comprising 14 four-point Likert-scaled items, seven for anxiety (HADS-Anxiety subscale) and seven for depression (HADS-Depression subscale). The HADS measures levels of symptoms in the last week. The total score of both subscales ranges from 0 to 21, with higher scores indicating more symptoms. Good mental functioning was defined as present when an individual had no anxiety (HADS-Anxiety subscale score < 8) and no depression (HADS-Depression subscale score < 8) [21].

Social functioning was assessed at follow-up using two subscales of the Maastricht Social Participation Profile (MSPP): consumptive participation (six items) and formal participation (three items) [22]. The MSPP measures frequency and diversity of social participation; the nine social activities included were defined by older people with a chronic disease themselves. The response categories of the MSPP range from 0 (‘not at all’) to 3 (‘more than twice a week’). A total score was calculated ranging from 0 to 27, with higher scores indicating more diverse or more frequent social participation. Good social functioning was defined as present when an individual had a score of ≥ 4 on the MSPP, which was the median in our study.

### Predictors of resilience

Predictors of resilience were measured at baseline and included: demographics (sex, age in years, educational level), personality (mastery), lifestyle (smoking, alcohol consumption, Body Mass Index, physical activity), social conditions (partner status, social contacts) and health (number of chronic diseases, grip strength). Measurement procedures were described elsewhere [17] and summarized below.

Educational level was classified into four categories: no education (elementary school not completed), low (elementary school completed), intermediate (vocational or general secondary education), or high education (college or university education).

Mastery, that is the extent to which individuals consider themselves to be in control of events and ongoing situations, was measured using a 6-item version of the Pearlin Mastery Scale [23]. The total scale score of the Pearlin Mastery Scale ranges from 6 to 30, with higher scores indicating a greater sense of mastery.

Smoking status (current or former versus never) and alcohol consumption (yes versus no) were assessed by self-report. Body Mass Index was calculated as weight in kilograms divided by height in meters squared. Weight was measured to the nearest 0.1 kg using a calibrated scale. Height was measured to the nearest 0.001 m using a stadiometer. Physical activity was measured using the LASA Physical Activity Questionnaire [24], which covers frequency and duration of various activities during the previous two weeks. Activities include walking outside, cycling, gardening, light and heavy household work and a maximum of two sports. In order to calculate the average daily physical activity in minutes per day, the frequency and duration of the individual activities were multiplied and divided by 14 days, and, subsequently, summed.

Partner status was assessed by asking whether participants were (1) single or never married, (2) married or cohabitating, (3) divorced, (4) widowed, or (5) in a registered partnership or living apart. In the analyses, partner status was dichotomized into having a partner (categories 2 and 5) versus not having a partner (categories 1, 3 and 4). The Lubben Social Network Scale was used to assess family (3 items) and friendship contacts (3 items) [25]. Responses for questions measuring number of contacts ranged from 0 (none) to 5 (nine or more). Total subscale scores were calculated ranging from 0 to 15, with higher scores indicating more contacts.

Number of chronic diseases was measured through self-reported presence of the following chronic diseases: chronic non-specific lung disease, cardiovascular diseases, peripheral artery diseases, diabetes mellitus, stroke, cancer, and osteoporosis. Grip strength was measured with a strain-gauged dynamometer. Participants were asked to perform two maximum force trails with each hand. To calculate the total score, the maximum values of the right and the left hand were summed, and divided by two. If only one hand could be used, the maximum value of that hand was taken.

### Statistical analyses

Baseline characteristics of individuals having LLOA with persistent severe pain, LLOA without persistent severe pain, and LLOA with missing data on pain were reported using descriptive statistics. Differences in means between individuals having LLOA with and without persistent severe pain were tested using Independent-Samples T Tests; differences in medians were tested using Mann–Whitney U tests; and differences in frequencies were tested using Pearson Chi-square Tests. Similar procedures were followed to compare persons with and without missing data.

We calculated the percentage of people with LLOA and persistent severe pain who showed good physical, mental and/or social functioning. Predictors of resilience, i.e. good versus less good functioning, were examined using univariable, binary logistic regression analyses. Because of the low number of people showing good functioning, we dichotomized all categorical predictor variables.

In the above analyses, the definition of persistent severe pain was based on the highest tertile at baseline and follow-up of our study sample. In a sensitivity analysis, we also studied persistent severe pain based on population-based normative values of the WOMAC. For this, we selected all persons with a pain score of ≥ 3.0, which was the 75th percentile for 70–74 year olds [26]. For this analysis, we translated the estimate of 3.0 on a 0–10-point scale in the study of Bellamy et al. to 30 on the 0–100 point scale used in the current study.

The analyses were weighted to adjust for differences in the distributions of age and sex across the samples of the six cohort studies. Weights were calculated per sex and per five-year age category, using the formula W = N_exp_/N_obs_, where N_exp_ is the number of individuals in a specific age/sex category in the European population and N_obs_ is the number of individuals in a specific age/sex category in the cohort [17]. In all analyses, a p-value below 0.05 was considered as statistically significant. All analyses were performed using IBM SPSS Statistics version 26.0.

## Results

At baseline, 638 individuals had LLOA, of which 463 individuals had clinical knee OA, 67 had clinical hip OA, and 103 had both clinical knee and clinical hip OA. Of the remaining five individuals, four individuals had clinical knee OA and missing information on clinical hip OA, and one individual had clinical hip OA and missing information on clinical knee OA. Out of 638 individuals having LLOA at baseline, 95 (valid percentage: 14.9%) individuals had persistent severe pain, 390 (61.1%) did not have persistent severe pain and 153 (24.0%) had missing data on pain at baseline and/or follow-up. Baseline characteristics of individuals in these three groups are described in Table 1. A significant difference was observed between the countries, with The Netherlands and Spain having the highest percentage of persons with persistent severe pain and Germany and Italy the lowest percentage of persons with persistent severe pain. Furthermore, persons with persistent severe pain had a significantly higher BMI and lower score on the Lubben Social Network Scale for family as compared with persons without persistent severe pain. No other meaningful differences between persons with and without persistent severe pain were observed. When comparing persons with and without missing data, persons with missing data were significantly older, were more often female, had lower mastery, less often a partner, more often one or more chronic diseases and a lower grip strength as compared with persons without missing data. Furthermore, there were country differences with the most missing data in Italy, which was the only country that started a new sample. Lifestyle and social network did not show differences between persons with and without missing data.

**Table 1:** Baseline characteristics of individuals with lower limb osteoarthritis and persistent severe pain (*n* = 638), weighted unless specified otherwise

	LLOA with persistent severe pain^a^	LLOA without persistent severe pain^a^	*P*-value^b^	LLOA with missing data on pain	*P*-value^c^
Number of individuals (unweighted)	95	390		153	
Age in years (unweighted)	74.6 ± 4.8	73.9 ± 5.0	0.237	75.9 ± 5.4	< 0.001*
Sex, women (%, unweighted)	69.5	63.1	0.243	76.5	0.005*
Country of residence (%)					
Germany	7.6	6.7	0.013*	6.5	< 0.001*
Italy	8.8	24.1		38.3	
the Netherlands	26.0	14.8		23.4	
Spain	23.8	22.9		15.3	
Sweden	19.9	20.6		11.7	
United Kingdom	13.9	10.9		4.8	
Educational level (%)					
No education	17.8	16.3	0.433	19.1	0.062
Low education	38.2	38.6		43.3	
Intermediate education	24.4	31.3		30.9	
High education	19.7	13.8		6.7	
Mastery	17.7 ± 6.0	18.2 ± 5.2	0.440	15.8 ± 5.0	< 0.001*
Smoking, current or former %)	48.6	46.7	0.692	38.1	0.055
Alcohol use, yes (%)	64.6	70.4	0.247	70.7	0.713
Body Mass Index (kg/m^2^)	30.6 ± 5.1	28.9 ± 4.7	0.002*	28.8 ± 5.0	0.405
Physical activity (min/day) ^d^	173.8 [97.2–220.5]	180.0 [101.7–255.9]	0.360	164.5 [90.0–278.6]	0.971
Partner status, % yes	59.9	68.2	0.141	56.9	0.029*
Lubben Social Network Scale (family)	8.0 ± 3.8	9.1 ± 3.2	0.012*	9.0 ± 3.3	0.893
Lubben Social Network Scale (friends)	7.2 ± 3.6	7.7 ± 3.5	0.239	6.7 ± 4.0	0.008*
Number of chronic diseases (%)					
0	23.8	33.9	0.070	16.3	< 0.001*
1	32.0	33.4		38.0	
2 or more	44.2	32.7		45.7	
Grip strength in kg	23.3 ± 9.5	24.7 ± 10.2	0.214	22.0 ± 9.9	0.011*

Presented are the mean and standard deviation unless stated otherwise. Differences in mean were tested using Independent-Samples T Tests; differences in median were tested using Mann–Whitney U test; differences in frequencies were tested using Pearson Chi-square Test

^a^Numbers can vary slightly between variables because of missing values

^b^Comparison: individuals with and without persistent severe pain

^c^Comparison: individuals with and without data

^d^Median [interquartile range]

*P-value < 0.05

Of the 95 individuals with LLOA and persistent severe pain, 10 (11.0%) had good physical functioning, 54 (57.4%) had good mental functioning, and 49 (53.8%) had good social functioning at follow-up. When looking at the number of domains, 19 (20.0%) individuals did not function well in any of the domains, 43 (45.3%) functioned well in one domain, 29 (30.5%) in two domains, and only 4 individuals (4.5%) were resilient in all three domains combined. During follow-up, one person reported a total knee prosthesis and no persons reported a total hip prosthesis.

In Table 2, predictors of good functioning are presented in individuals having LLOA with persistent severe pain. Higher physical activity levels were associated with higher odds on good physical functioning (Odds Ratio (OR) = 1.01 for a 1-min/day higher physical activity level; 95% Confidence Interval (CI) = 1.00–1.01). People with intermediate or high education as compared to lower education (OR = 2.50; 95% CI = 1.04–6.01) and people with a 1-point higher level of mastery (OR = 1.22; 95% CI = 1.10–1.35) had higher odds on good mental functioning. In addition, people with one or more chronic diseases had lower odds on good mental functioning as compared with people without diseases (OR = 0.29; 95% CI = 0.09–0.90). Higher age (OR = 0.90 for a 1-year higher age; 95% CI = 0.82–0.99) and female sex (OR = 0.27; 95% CI = 0.10–0.72) were significantly related to lower odds on good social functioning. People with intermediate or high education (OR = 2.49; 95% CI = 1.06–5.89), reporting to smoke or having smoked in the past (OR = 2.50; 95% CI = 1.06–5.89), reporting alcohol use (OR = 3.35; 95% CI = 1.34–8.38) and people with a 1-point higher score on the Lubben Social Network Scale for friends had higher odds on good social functioning (OR = 1.32; 95% CI = 1.15–1.53).

**Table 2:** Predictors of resilience, i.e. good physical, mental or social functioning in older adults with lower limb osteoarthritis and persistent severe pain (*N* = 95), weighted unless specified otherwise

	**OR (95% CI) on good versus less good physical functioning**	**OR (95% CI) on good versus less good mental functioning**	**OR (95% CI) on good versus less good social functioning**
Age (unweighted)	0.90 (0.77–1.05)	0.95 (0.87–1.04)	0.90 (0.82–0.99)*
Female sex (unweighted)	0.40 (0.11–1.50)	1.02 (0.42–2.49)	0.27 (0.10–0.72)*
Intermediate or high versus low or no education	3.21 (0.68–15.13)	2.50 (1.04–6.01)*	2.49 (1.06–5.89)*
Mastery	1.13 (0.99–1.30)	1.22 (1.10–1.35)*	1.04 (0.97–1.12)
Current or former smoker versus never smoker	0.31 (0.06–1.58)	1.17 (0.50–2.71)	2.50 (1.06–5.89)*
Alcohol use: yes versus no	^a^	1.66 (0.69–3.97)	3.35 (1.34–8.38)*
Body Mass Index (kg/m^2^)	0.92 (0.79–1.08)	0.98 (0.90–1.06)	1.01 (0.93–1.09)
Physical activity (min/day)^b^	1.01 (1.00–1.01)*	1.00 (1.00–1.01)	1.00 (1.00–1.01)
Having a partner versus not having a partner	1.13 (0.26–4.88)	1.50 (0.64–3.50)	0.71 (0.30–1.66)
Lubben Social Network Scale (family)	1.10 (0.91–1.34)	1.08 (0.96–1.21)	1.03 (0.92–1.15)
Lubben Social Network Scale (friends)	1.23 (0.98–1.54)	1.12 (1.00–1.27)	1.32 (1.15–1.53)*
One or more chronic diseases versus no chronic disease	0.83 (0.17–4.17)	0.29 (0.09–0.90)*	1.07 (0.40–2.87)
Grip strength	1.06 (0.99–1.14)	1.02 (0.98–1.07)	1.04 (0.99–1.08)

OR=odds ratio; 95% CI=95% confidence interval

^a^Not possible to perform logistic regression analysis as all individuals consumed alcohol.

^*^P-value<0.05

In a sensitivity analysis, we also studied persistent severe pain using a threshold of ≥ 30 to define persistent severe pain, which is the 75^th^ percentile of 70–74 year old individuals in a population-based study that aimed to estimate norm values [26]. In total, 146 persons (30.0%) out of 638 individuals with LLOA had persistent severe pain. Among these, 21 (14.8%) had good physical functioning, 92 (63.4%) had good mental functioning, and 75 (54.0%) had good social functioning. Only 10 individuals (7.4%) were resilient in all three domains of functioning. These results are largely in line with our main results.

## Discussion

In this prospective cohort study in a representative sample of community-dwelling older adults across six European countries, 95 out of 638 individuals with LLOA had persistent severe pain. From these, 11.0% had good physical functioning, 57.4% had good mental functioning, 53.8% had good social functioning at follow-up, and only 4.5% were resilient in all three domains combined. To our knowledge, this is the first study applying the concept of resilience to individuals having LLOA with persistent severe pain while assessing three important domains of functioning. It is concerning that 14.9% report persistent severe pain and that only few of these people had good physical functioning and that in almost half of them mental and social functioning was negatively affected.

To our knowledge, no other studies examined good physical, mental or social functioning despite having persistent severe pain, i.e., resilience, in persons with lower limb OA, which makes comparison with the literature difficult. However, our results are largely in line with literature studying predictors of good or poor functioning in older adults regardless of OA. In our study, higher physical activity levels predicted good physical functioning, which is in line with an earlier study in which more time spent in habitual light-intensity physical activity was associated with better lower-extremity performance in community-dwelling older adults [27]. In another study, low physical activity was associated with the development of functional limitations in adults with knee OA [28]. Furthermore, Kraus et al. found strong evidence that physical activity improved physical function among individual with hip or knee OA relative to less active adults with OA [29]. In our study, higher education, higher mastery and absence of chronic disease were related to good mental functioning. This is in line with a study in which a higher educational level was associated with better psychological well-being in persons aged 50 years or older [30]. In another study, lower educational level, lower mastery and a higher number of chronic diseases were associated with onset of depressive symptoms and comorbid anxiety-depression, but not with anxiety alone [31]. In a study by Seo and colleagues, the presence of two or more chronic diseases was associated with depressive symptoms in older adults [32]. Finally, younger age, male sex, higher education, smoking and alcohol use and more contacts with friends predicted good social functioning in our study. These findings are in line with data from the MoPAct project (i.e., Mobilising the potential of active ageing in Europe) showing that both in multimorbid and non-multimorbid groups, and across different types of participation, a higher socioeconomic status and larger social network were associated with higher levels of participation [33]. In addition, using data from the Survey of Health, Ageing, and Retirement in Europe, younger age, male sex and higher education were associated with volunteering work [34]. In the Longitudinal Aging Study Amsterdam, higher education and a large social network increased the likelihood of volunteering [35].

Interestingly, only 14.9% of our sample with LLOA had persistent severe pain when using the highest tertile of the WOMAC pain score to define persistent severe pain. In a sensitivity analysis, we also studied persistent severe pain using population-based estimated norm values [26]. When using the 75^th^ percentile of 70–74 year old individuals, 30.0% of individuals with LLOA had persistent severe pain. As these are population-based norm values including both healthy persons and persons with one or more comorbidities, more persons fulfill the definition of persistent severe pain than when using a stricter threshold. In addition, it should be noted that these normative values are based on an Australian sample, which may not directly translate to our European sample.

When looking at the characteristics of persons with and without persistent severe pain, it was found that persons with persistent severe pain had a significantly higher BMI and lower score on the Lubben Social Network Scale for family as compared with persons without persistent severe pain. This is in line with earlier research. In a random sample of 5000 Australians, it was found that in persons with arthritis or OA, obese individuals reported more hip and knee pain [36]. Furthermore, in another study, family support was associated with less pain in persons with arthritic conditions [37]. However, the latter result was not statistically significant.

Our findings indicate that more attention is needed for older adults with LLOA who have persistent severe pain as only few persons with LLOA and persistent severe pain showed good functioning, especially with regard to physical functioning, but also with regard to social and mental functioning. Their treatment should particularly focus on reducing pain and on improving functioning. Recommendations already exist for adequate pain management in individuals with clinical OA, including education and self-management programs, weight management, general exercise, strength and resistance training, and adequate pharmacological treatment [38]. As pain has a negative impact on physical functioning [39, 40] and some of the above interventions directly improve physical performance, reducing pain might have positive effects on functioning as well. As an alternative approach, interventions aimed at improving functioning by intervening on predictors related to resilience might yield positive results. In the current study, a wide range of potential predictors of resilience were explored. Among these predictors, only higher physical activity levels were related to good physical functioning, which fits to earlier recommendations promoting general exercise [38, 41]. Higher age and female sex were related to lower odds of good social functioning, and intermediate to high education was related to higher odds on good mental or social functioning. As these predictors are non-modifiable, they may be used to identify individuals at high risk of low functioning. Some of the other findings may serve as a basis for the development of new interventions or reuse and adaptation of existing interventions. In particular, enhancement of sense of mastery and contacts with friends seem promising elements of such interventions. A promising intervention affecting these outcomes might be the Chronic Disease Self-Management Program [42, 43].

A strength of this study is that six European cohorts with representative samples of community-dwelling older adults were studied with standardized data collection procedures. Another strength is the focus on resilience instead of negative outcomes, and the wide range of both predictors and outcomes that were studied. A weakness is the relatively small sample of 95 individuals in which the predictors of resilience were studied, which is a drawback of using a representative sample of community-dwelling older adults, where only a fraction is affected by LLOA and has persistent severe pain. An advantage, however, is that the current study gives more insight in the actual number of people affected and the number of people showing resilience in the general population. Because of the small sample size, we chose the median as a statistical cut-off point for good physical and social functioning. In future research it might be good to choose a stricter cut-off point, indicating optimal functioning. Finally, it would have been interesting to have information about the stage of OA. As only people with persistent severe pain were included in our sample, we assume that our sample does not include people with early stage OA.

## Conclusions

In conclusion, in individuals having LLOA with persistent severe pain, only few individuals showed good physical functioning and about half of participants showed good mental or social functioning. Predictors of resilience differed between domains of functioning, and might provide new insights for treatment.

## Data Availability

The data that support the findings of this study are available from https:// www.eposa.org/ but restrictions apply to the availability of these data, which were used under license for the current study, and so are not publicly available. Data are however available from the authors upon reasonable request and with permission of the EPOSA Research Group.

## Abbreviations

CI: Confidence Interval
EPOSA: European Project on OSteoArthritis
HADS: Hospital Anxiety Depression Scale
IBM SPSS: International Business Machines Corporation Statistical Package for the Social Sciences
kg: Kilogram
kg/m^2^: Kilogram per square meter
LASA: Longitudinal Aging Study Amsterdam
LLOA: Lower limb osteoarthritis
Min/day: Minutes per day
MoPAct: Mobilising the potential of active ageing in Europe
MSPP: Maastricht Social Participation Profile
n: Number
N_exp_: Number of individuals in a specific age/sex category in the European population
N_obs_: Number of individuals in a specific age/sex category in the cohort
OA: Osteoarthritis
OR: Odds ratio
W: Weight
WOMAC: Western Ontario and McMaster Universities Osteoarthritis Index
